# Timing and risk factors associated with acute kidney injury in infants with necrotizing enterocolitis

**DOI:** 10.1038/s41372-024-02003-4

**Published:** 2024-05-22

**Authors:** Geethanjali Lingasubramanian, Christina Eva Hum, Krystal Hunter, Rajeev Mehta, Vineet Bhandari

**Affiliations:** 1 Rutgers Robert Wood Johnson Medical School, New Brunswick, NJ USA; 2 Cooper Medical School of Rowan University, Camden, NJ USA; 3 The Children’s Regional Hospital at Cooper, Camden, NJ USA

**Keywords:** Risk factors, Acute kidney injury

## Abstract

**Objective:**

To evaluate the prevalence, onset, risk factors, and mortality associated with acute kidney injury (AKI) in infants with necrotizing enterocolitis (NEC).

**Design/Methods:**

Retrospective study at 2 centers in infants with NEC, with/without AKI. AKI assessed by serum creatinine and urine output. Statistical tests used included t, Mann-Whitney U, Chi-square, and Fisher Exact tests.

**Results:**

Among 80 eligible infants with NEC, 56 (70%) had AKI. Median onset of NEC was day 15, with median AKI onset two days (IQR, –5.75 to 0) prior to NEC onset. Vasopressors were significantly more likely to be used in infants with NEC and AKI (*p* = 0.009). Increased mortality (*p* = 0.01) was noted in infants with NEC and AKI.

**Conclusions:**

The onset of AKI mostly precedes NEC onset, with moderate to severe AKI more prevalent than the milder form in infants with NEC. These infants are significantly more likely to be hemodynamically unstable and have increased mortality.

## Introduction

Necrotizing enterocolitis (NEC) remains a leading cause of morbidity and mortality among premature neonates [1–3]. NEC mortality rates range from 15% to 30%, with smaller infants [4] with more severe disease and those requiring surgery [5, 6] having higher case fatality rates. Survivors of NEC are at increased risk for adverse neurodevelopment outcomes [7, 8]. Despite decades of research, the pathogenesis of this disease remains unclear and is likely multi-factorial. Prevention and treatment strategies are inadequate and evolving. Hence, it is imperative to identify the potential risk factors to mitigate this problem.

One risk factor identified is acute kidney injury (AKI) which is associated with adverse outcomes in neonates with NEC [9, 10]. However, very few studies have explored this association and have reported varying results [11–15]. Studies have shown an increase in mortality rate and length of stay (LOS) in neonates with NEC who develop AKI [11–14]. In most of these studies, screening for AKI was done on, or after the onset of NEC [11–14], and AKI diagnosis was based on serum creatinine measurements only [11, 12, 15]. Surgical NEC, blood culture-positive sepsis, and nephrotoxic medications were some risk factors identified in the association of NEC with AKI [12, 14].

This study aims to evaluate the prevalence and onset of AKI in infants with NEC and the risk factors involved in the association of AKI with NEC. We hypothesized that infants affected with NEC have unique risk factors predisposing them to AKI.

## Methods

### Study design

This is a retrospective cohort study conducted at two centers in New Jersey - The Children’s Regional Hospital at Cooper/Cooper University Hospital (CUH) and Robert Wood Johnson University Hospital (RWJ). Both are Level IIIB urban in-born and referral neonatal intensive care units (NICUs). This study was approved by the Institutional Review Boards at both institutions. Data were collected from January 2011 to December 2022. Infants <32 weeks gestational age (GA) and/or birth weight (BW) < 1500 gm with NEC diagnosis (as per Modified Bell’s staging ≥ Stage 2) [16] were included in this study. Infants with clinical/stage 1 NEC, spontaneous intestinal perforation, and missing clinical data were excluded from this study.

### Demographics

We collected demographic data, which included GA, sex, BW, and race. We collected data on outborn status, mode of delivery, Apgar scores at 5 min, and presence of perinatal asphyxia. Maternal variables such as pregnancy-induced hypertension (PIH), chorioamnionitis, Diabetes mellitus, and antenatal steroids were also collected.

### NEC variables

We recorded the age of the infant in days at the onset of NEC, the presence of pneumatosis, portal venous gas, or pneumoperitoneum in abdominal X-rays read by a radiologist, and the placement of a peritoneal drain or laparotomy performed for the treatment of NEC. NEC onset was defined as the day of life of infant when the NEC diagnosis was first made, as per the clinical team, based on their documentation.

### AKI variables

Baseline serum creatinine (SCr), the lowest serum creatinine noted from birth till one week before the onset of NEC, was recorded. We also recorded the day of life (DOL) when serum creatinine was the lowest. Then, serum creatinine was trended one week before and one week after the onset of NEC for 14 days. Urine output was also trended during this period which was documented in electronic medical records in 24-hour blocks and calculated in ml/kg/hour. Urine output was measured by weighing diapers. Foley catheters were not used. Infants who meet the modified KDIGO criteria [17] were diagnosed with Stage 1 AKI if there is a rise in SCr by 0.3 mg/dL or a rise by 1.5–1.9 times above baseline and/or urine output (UOP) < 1 mL/kg/h over the last 24 h, Stage 2 AKI if there was an increase in SCr 2–2.9 times above baseline and/ or UOP < 0.5 mL/kg/h, Stage 3 AKI if there was an increase in SCr three times above baseline or SCr >2.5 mg/dL and/or UOP < 0.3 mL/kg/h. We recorded the infant’s age in days at the onset of AKI, the staging of AKI at the onset and the maximum stage reached in the 14 days around the onset of NEC. We used the maximum stage to calculate the incidence of AKI severity in our cohort.

### Hemodynamic variables

We collected information on patent ductus arteriosus (PDA; confirmed by echocardiography), treatment for PDA with indomethacin, ibuprofen, congenital heart disease, and vasopressor use in the 14 days around NEC onset.

### Other variables

We collected data on nephrotoxic antibiotics such as gentamicin and vancomycin used from birth till one week after NEC onset, blood culture-positive sepsis, and diuretics usage. We did not specifically collect data on caffeine; however, it is standard practice in our NICUs to use caffeine in all infants with these gestational ages, till they reach at least 32-34 weeks of gestation for consideration of stoppage of caffeine.

Demographics and clinical variables were compared between the two groups of infants with NEC, with and without AKI.

### Statistical analysis

Data for continuous variables with normal distribution were expressed as mean and standard deviations, and comparisons between those with AKI and without AKI were performed using the Independent t-test. Mann-Whitney U test was used for continuous variables with skewed distribution and data was expressed as the median and interquartile range (IQR). Analysis was done using Chi-squared or Fisher’s exact test for categorical variables and data expressed as percentages. Logistic regression analysis was used for AKI and mortality outcomes. Clinical variables (based on earlier reports on this topic) that could potentially impact the presence of AKI in NEC and mortality were chosen for this analysis, even when statistically non-significant, based on the reviewer’s request. These variables are expressed as odds ratio with 95% confidence intervals. A *p* value < 0.05 was considered statistically significant for all the analyses.

### Outcomes

Primary outcomes assessed were mortality rate and LOS in infants with NEC with or without AKI.

## Results

**Fig. 1:**
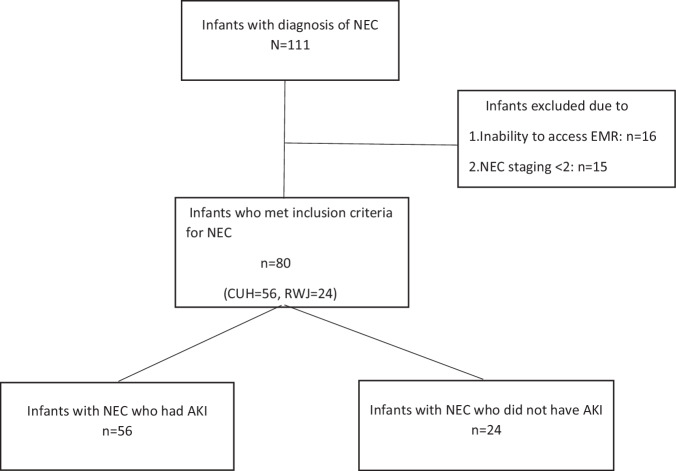
Flow diagram of our study cohort. NEC necrotizing enterocolitis, CUH The Children’s Regional Hospital at Cooper/Cooper University Hospital, RWJ Robert Wood Johnson University Hospital, AKI acute kidney injury, EMR electronic medical records.

Among 111 infants diagnosed with NEC, 80 met inclusion criteria, and 31 were excluded due to inability to access medical records or NEC staging <2 (Fig. 1).

### NEC with AKI

Among 80 eligible infants with NEC, 56 (70%) had AKI of any stage, and 24 (30%) did not have AKI. Nine (16.1%) had stage 1, 23 (41.1%) had stage 2, and 24 (42.9%) had stage 3 AKI. A comparison of the NEC infants with or without AKI is shown in Table 1.

**Table 1:** Characteristics of infants with NEC, with and without AKI.

Variables	Combined cohort *N* = 80	NEC without AKI *N* = 24	NEC with AKI *N* = 56	*p* value
Gestational age (mean, SD)	27.02 (2.78)	26.79 (2.39)	27.12 (2.94)	0.621
Male Sex (*n*, %)	48 (60)	13 (54.2)	35 (62.5)	0.486
Black race (*n*, %)	42 (52.5)	16 (66.7)	26 (46.4)	0.097
Birth weight (mean, SD)	841.63 (288.31)	846.92 (260.11)	839.36 (301.80)	0.915
C-section (*n*, %)	36 (45)	11 (45.8)	25 (44.6)	0.922
APGAR at 5 min < 7 (*n*, %)	28 (35)	11 (45.8)	17 (30.3)	0.184
Clinical Chorioamnionitis (*n*, %)	8 (10)	1 (4.2)	7 (12.5)	0.424
Pregnancy-induced hypertension (*n*, %)	25 (31.2)	8 (33.3)	17 (30.4)	0.792
Antenatal steroids (*n*, %)	74 (92.5)	22 (91.7)	52 (92.9)	1.000
Age at NEC onset, median (IQR)	15 (10–27)	15.5 (8.25-33.5)	15 (10-26.25)	0.757
Medical NEC (stage 2) (*n*, %)	38 (47.5)	12 (50)	26 (46.4)	0.884
Surgical NEC (*n*, %)	42 (52.5)	12 (50)	30 (53.6)	0.769
Blood transfusion (*n*, %)	72 (90)	22 (91.7)	50 (89.3)	1.000
Patent Ductus Arteriosus (*n*, %)	38 (47.5)	12 (50)	26 (46.4)	0.884
Use of Vasopressors (*n*, %)	59 (73.7)	13 (54.2)	46 (82.1)	0.009
Sepsis (Blood culture positive) (*n*, %)	41 (51.3)	11 (45.8)	30 (53.6)	0.526
Gentamicin (*n*, %)	79 (98.8)	24 (100)	55 (98.2)	1.000
Vancomycin (*n*, %)	64 (80)	18 (75)	46 (82.1)	0.545
Length of stay in survivors, days median (IQR)	102.5 (62.25–130.75) *N* = 46	116 (70–133) *N* = 19	88 (51–130) *N* = 27	0.269
Death (*n*, %)	34 (42.5)	5 (20.8)	29 (51.8)	0.010

### Onset of NEC and AKI

The median onset of NEC was 15 days, and the median AKI onset was two days prior to the onset of NEC (interquartile range –5.75 to 0 days) i.e., 13 days (IQR 9 to 15days).

### Hemodynamic variables

Among hemodynamic variables, vasopressors use was significantly higher in infants with NEC associated with AKI 82.1% vs. 54.2% (*p* = 0.009; Table 1). There was a trend towards higher dobutamine use among infants with NEC who had AKI compared to infants without AKI requiring vasopressors at 51.9% vs. 26.3% (*p* = 0.055).

### Other variables

There was no statistically significant difference in demographics, antenatal, NEC variables or nephrotoxic antibiotics use between the two groups. There were no outborn infants in the whole cohort.

### Mortality rate

Among 56 infants with NEC who had AKI, 29 (51.8%) died, whereas five infants out of 24 (20.8%) died in the other group (*p* = 0.010; Table 1).

### Length of stay

The overall median LOS in all subjects was 70 days (IQR 30-116). Among the survivors, the median LOS in NEC with AKI group was 88 days (IQR 51–130) when compared to infants with NEC who did not have AKI 116 days (IQR 70–133) *p* = 0.269 (Table 1).

### Surgical NEC

Twenty-two infants out of 29 who died had surgical NEC with AKI, accounting for a mortality rate of 75.8%. Among 47 infants with NEC with severe AKI (stage 2 and 3), 36 (76%) had surgical NEC.

### Blood culture-positive sepsis

There was no statistically significant difference in blood culture positive sepsis between the two groups 53.6% vs. 45.8% (*p* = 0.526). Of the 41 infants with NEC and blood culture-positive sepsis, 30 (73%) developed AKI, and 17 (41.1%) died.

### AKI diagnosis

Among the 56 infants who had AKI, 27 (48.2%) were diagnosed with AKI based on both SCr and UOP criteria, 23 (41%) based on SCr only, and 6 (10.7%) based on UOP only (Table 2). Of the 27 infants diagnosed with AKI based on SCr and UOP, 13 (48%) had higher staging with UOP criteria. Six of 13 (46%) infants with higher staging based on UOP were diagnosed with AKI 1–2 days before being diagnosed with elevated SCr. Seven out of 56 infants with AKI progressed from stage 1 to 3 in the 14 days around the onset of NEC. Duration noted for progression of AKI from stage 1 to 3 was 1–3 days based on UOP criteria and 4–8 days based on SCr criteria. The baseline SCr in this cohort was 0.4 (0.2–0.7). The lowest SCr prior to NEC onset was noted to be at a median age of 5 days. Ten out of 56 infants (18%) with AKI had a previous episode of AKI. No infants were treated with renal replacement therapy.

**Table 2:** Diagnosis and staging of AKI based on Kidney Disease: Improving Global Outcomes (KDIGO) criteria.

AKI Stages	Serum creatinine	Urine output	Serum creatinine + urine output	Total
Stage 1	7	1	1	9
Stage 2	8	2	13	23
Stage 3	8	3	13	24
Total	23	6	27	56

Logistic regression analyses showed the use of vasopressors increases the odds of AKI (OR 8.18, 95% CI 1.82–36.85) and mortality (OR 31.73, 95% CI 3.13–321.83). Otherwise, there was no statistically significant difference in demographics, antenatal, or other variables between the two groups (Table 3A, B).

**Table 3:** **A** Logistic regression outcome AKI—Full sample. **B** Logistic regression outcome mortality—Full sample.

Variables	OR	95% C.I.	*p* value
**A**			
Gestational age	1.181	0.926–1.507	0.180
Gender-Male	1.988	0.618–6.391	0.249
Race-Black	0.550	0.174–1.740	0.309
Apgar score at 5 min	1.183	0.850–1.648	0.320
Chorioamnionitis	3.473	0.304–39.633	0.316
Antenatal steroids	1.410	0.191–10.429	0.736
Vasopressors	8.182	1.817–36.852	0.006
Vancomycin	1.257	0.302–5.239	0.754
Sepsis	0.669	0.198–2.267	0.519
**B**			
Gestational age	1.168	0.911–1.499	0.221
Gender-Male	1.214	0.360–4.092	0.754
Race-Black	1.994	0.624–6.377	0.244
Apgar score at 5 min	0.940	0.660–1.340	0.734
Chorioamnionitis	6.723	0.686–65.882	0.102
Antenatal steroids	3.699	0.344–39.824	0.281
Vasopressors	31.730	3.128–321.825	0.003
Vancomycin	1.184	0.234–5.991	0.839
Sepsis	1.371	0.390–4.817	0.622
Acute Kidney Injury	2.753	0.717–10.563	0.140

### Results from the two centers

The patient population from Robert Wood Johnson University Hospital and The Children’s Regional Hospital at Cooper were similar in terms of demographics, antenatal, NEC, AKI and hemodynamic variables. Median onset of NEC was similar and AKI onset was on or before the onset of NEC in both the groups. The mortality rate in infants with NEC who have AKI was also similar at 50% and 52.5% respectively.

In the Robert Wood Johnson University Hospital cohort, there was statistically significant difference in mortality rate between the groups of infants with NEC with and without AKI. (*p* = 0.022) whereas the cohort from The Children’s Regional Hospital at Cooper had statistically significant difference in vasopressors use among infants having NEC with AKI (*p* = 0.035) (Please refer to Supplemental Information including Supplemental Table 1–7 for details).

## Discussion

This study showed that the median onset of NEC was 15 days, and the median AKI onset in infants with NEC was two days prior to the onset of NEC (interquartile range -5.75 to 0 days) i.e. 13 days (IQR 9 to 15days). Vasopressor use was significantly higher in infants with NEC associated with AKI 82.1% vs. 54.2% (*p* = 0.009; Table 1). Moderate to severe AKI more prevalent than the milder form in infants with NEC and have an increased mortality rate.

Previous studies [11–14] have shown the association of AKI that develops after NEC onset. Various pathophysiologic explanations were given for this association, such as a significant inflammatory cascade caused by NEC that can lead to microcirculatory disturbance, sepsis causing hypotension or direct tubular damage, nephrotoxic medications used after NEC diagnosis [18, 19]. Accordingly, it is presumed that AKI develops after NEC onset, and previous studies have focused on AKI onset on or after NEC onset. In contrast to these studies, this study shows that the onset of AKI precedes the week before the onset of NEC. This critical finding questions our understanding of the pathophysiology involved in the association of NEC with AKI.

This study also showed that a significant proportion of infants with NEC who have AKI required vasopressors indicating that these infants were hemodynamically unstable. This finding invokes an exciting hypothesis that impaired perfusion due to hemodynamic instability could be a common factor in the development of AKI and NEC. Studies have shown that intestinal microcirculatory dysfunction plays a significant role in the pathogenesis of NEC [20]. Animal models show similar pathophysiology in both AKI and NEC. The cecal ligation and puncture AKI animal model has many features similar to NEC. Studies also suggest a causal relationship in which AKI appears to drive other organ dysfunction and vice-versa, referred to as “crosstalk.” [21–23]. Thus, systemic inflammation associated with AKI could affect the intestines and cause NEC.

AKI incidence in infants with NEC in this study was found to be 70% which is higher than the reported incidence of 21–54% in the previous studies. This could be due to the increased recognition of AKI that was prevalent the week before the onset of NEC and by evaluating both SCr and UOP for AKI diagnosis. The other possibility for higher incidence could be from previous episodes of AKI, which predisposes to recurrent AKI. In a retrospective report from the AWAKEN database, in infants with late-onset AKI ( > 7 days of age), 28% had an earlier episode of AKI [24]. We explored this option and found that in our cohort, we had 18% with previous AKI, which is less likely to explain the higher incidence in this cohort.

This study also showed that most infants with NEC and AKI progressed to moderate-severe AKI. The incidence of severe AKI (stages 2 and 3) in this cohort was 84%, and 76% had surgical NEC. This is a significant finding compared to the study by Garg et al. [14], who reported 32.6% incidence of severe AKI; 58.7% after surgical NEC, despite a similar incidence rate of surgical NEC in both studies (52% vs. 51.5%) respectively. Again, this is possibly due to increased recognition of AKI and a quicker progression to severe stages before NEC onset.

Most previous studies have utilized SCr only for AKI diagnosis, except for Garg et al., who monitored SCr and UOP per KDIGO criteria. In our study, 10.7% of infants with AKI were diagnosed by UOP criteria alone. Moreover, among infants diagnosed with AKI based on both SCr and UOP, 48% of infants had higher staging with UOP criteria. The progression of AKI severity is also quicker when UOP criteria are followed for AKI diagnosis, thus stressing the importance of including UOP measurements also in AKI diagnosis along with SCr.

This study is in concordance with the other studies [11–14], which showed an increase in mortality rate among infants with NEC associated with AKI. We found a two-fold increase in mortality rate in infants with NEC with AKI compared to those without AKI, confirming the findings reported by Criss et al. [11]. The mortality rate was even higher, up to 75.8% in infants with surgical NEC with AKI. We did not find any statistically significant difference in the LOS among the survivors, between the two groups.

In a recent study, Han et al. investigated the potential use of changes in SCr, reflecting the development of AKI as a surrogate biomarker for the impending development of NEC [15]. They evaluated AKI in preterm infants born between 23- and 32 weeks GA who did and did not develop NEC and the incidence of AKI in infants one week before the diagnosis of medical and surgical NEC. AKI was screened using SCr measurements only from the DOL 8 onwards. They reported that one of the 13 with AKI had AKI within 7 days before NEC diagnosis (7.7%). Our study is similar to the above in evaluating AKI onset one week before NEC diagnosis but differs in several other ways. Our cohort consists of infants with NEC, and a comparison was made between those with or without AKI. Both SCr and UOP criteria were used for AKI diagnosis, and we also included SCr measurements from the first week of life. Out of 56 infants with AKI, 47 (84%) had AKI within 7 days before NEC onset. We speculate that this significant difference in results from Han et al. is due to the difference in AKI diagnostic criteria and patient population.

The mean GA in this cohort was 27 weeks, with a mean BW of ~840 g, and the median age at NEC onset was 15 days. According to a study by Yee et al., preterm infants with BW > 1000 grams present with NEC at a mean of 7 days (early onset) versus a mean of 32 days (late onset) in infants with BW < 1000 g [25]. In comparison to this study, NEC onset seems to be earlier in our study. It is unclear whether this earlier onset of NEC affects the association with AKI.

We found similar results in the two centers where we conducted this study, such as median NEC and AKI onset, prevalence, and severity of AKI, which emphasizes the credibility of this finding.

### Limitations

Since this is a retrospective study, cause-and-effect relationship claims cannot be made. The small sample size makes it difficult to generalize the study results. SCr is a marker of renal function and not renal injury, so elevated SCr merely reflects a decline in renal function. SCr varies with gestational and postnatal age. In preterm infants, the decline in SCr after birth is slower. It reaches nadir values in the first 1–2 months, so identifying a baseline SCr value is challenging in the first month of life. Accurate UOP measurements were not always possible as these measurements are obtained by weighing diapers. However, this was true across both sites and study cohorts. Despite this, we used the KDIGO definition because it is the most widely accepted definition used by previous studies on NEC with AKI. It also allowed us to compare our study results.

This study is the first to evaluate the prevalence and timing of AKI one week before and after NEC onset, using SCr and UOP measurements. Regardless of the possible mechanisms involved in the association of NEC and AKI, as discussed above, the detection of AKI seems to precede NEC. In our cohort, the median age of NEC onset was 15 days, and infants had frequent electrolyte measurements, including SCr, in this period after birth and so we had adequate SCr measurements to trend and screen for AKI. This vital knowledge could help us with the early identification and management of NEC and prevent complications. Thus, close monitoring of SCr and UOP measurements is imperative in premature infants.

## Conclusion

This study brings a new perspective on this topic by the finding that AKI mostly precedes NEC onset. AKI incidence and severity are much higher than previously reported. These infants are significantly more likely to be hemodynamically unstable, requiring vasopressors with a significant increase in mortality rate.

## Supplementary information

Supplemental information

## Data Availability

The data from the individual sites presented in the study are included in the supplemental information; further inquiries can be directed to the corresponding author.
