# Supplementary material for: Timing and risk factors associated with acute kidney injury in infants with necrotizing enterocolitis
Source: J Perinatol. 2024 May 22;44(12):1774–9. doi: 10.1038/s41372-024-02003-4 (PMC11606910; doi:10.1038/s41372-024-02003-4)
Supplement: Supplementary file 1 — Supplemental information [file 41372_2024_2003_MOESM1_ESM.docx]

**SUPPLEMENTARY INFORMATION**

**Results from CUH:**

Among 72 infants diagnosed with NEC, 56 met the inclusion criteria, and 16 were excluded due to inability to access medical records. Among 56 eligible infants with NEC, 40 (71%) had AKI of any stage, and 16 (29%) did not have AKI. Six (15%) had stage 1, 17 (42.5%) had stage 2, and 17 (42.5%) had stage 3 AKI. **Supplemental Table 1** shows the comparison of NEC infants with or without AKI. AKI onset coincided or occurred prior to the onset of NEC (median AKI onset was one day before the onset of NEC, interquartile range -3 to 0 days). Vasopressors were significantly more likely to be used in infants having NEC with AKI (p=0.035; **Supplemental Table 1**). The groups had no statistically significant difference in mortality rate or LOS.

Among the 40 infants who had AKI,22 (55%) were diagnosed with AKI based on both SCr and UOP criteria, 12(30%) based on SCr only, and 6(15%) based on UOP only **(Supplemental Table 2)** Of the 22 infants diagnosed with AKI based on both SCr and UOP, 13 (59%) had higher staging with UOP criteria. Six of 13 (46%) infants with higher staging based on UOP were diagnosed with AKI 1-2 days before being diagnosed with elevated SCr. Duration noted for progression of AKI from stage 1 to 3 was 1-3 days.

Logistic regression analyses showed the use of vasopressors increases the odds of AKI (OR 9.02, 95% CI 1.33 - 61.03) and mortality (OR 19.13, 95% CI 1.67 - 219.05) **(Supplemental Tables 3 and 4).** Otherwise, there was no statistically significant difference in demographics, antenatal, or other variables between the two groups.

**Supplemental Table 1. Characteristics of infants with NEC, with and without AKI in the CUH cohort**

| Variables | NEC without AKI  N=16 | NEC with AKI  N=40 | p-value |
| --- | --- | --- | --- |
|  |  |  |  |
| **Gestational age (mean, SD)** | 27.2 (2.5) | 27.3 (3.2) | 0.906 |
| **Male Sex (n, %)** | 10 (62.5) | 27 (67.5) | 0.721 |
| **Black race (n, %)** | 12 (75) | 21 (52.5) | 0.122 |
| **Birth weight (mean, SD)** | 926 (276) | 837 (327) | 0.342 |
| **C-section (n, %)** | 3 (18.8) | 13 (32.5) | 0.350 |
| **APGAR at 5min <7 (n, %)** | 7(43.8) | 10 (25) | 0.168 |
| **Clinical Chorioamnionitis (n, %)** | 1 (6.3) | 5 (12.5) | 0.662 |
| **Pregnancy-induced hypertension (n, %)** | 4 (25) | 10 (25) | 1.000 |
| **Antenatal steroids (n, %)** | 16 (100) | 37 (92.5) | 0.550 |
| **Age at NEC onset, median (IQR)** | 15.5 (8.75-28.25) | 15.5 (10.25-28.5) | 0.884 |
| **Medical NEC (stage 2) (n, %)** | 8 (50) | 20 (50) | 1.000 |
| **Surgical NEC (n, %)** | 8 (50) | 20 (50) | 1.000 |
| **Blood transfusion (n, %)** | 14 (87.5) | 35 (87.5) | 1.000 |
| **Patent Ductus Arteriosus (n, %)** | 7 (43.8) | 19 (47.5) | 0.799 |
| **Use of Vasopressors (n, %)** | 9 (56.3) | 34 (85) | 0.035 |
| **Sepsis (Blood culture positive) (n, %)** | 9 (56.3) | 25 (62.5) | 0.665 |
| **Gentamicin (n, %)** | 16(100) | 40(100) | 1.000 |
| **Vancomycin (n, %)** | 14(87.5) | 36(90) | 1.000 |
| **Length of stay in survivors, days**  **median (IQR)** | 109(54-144) | 79(43-130) | 0.372 |
| **Death (n, %)** | 5 (31.3) | 21 (52.5) | 0.150 |
|  |  |  |  |

**Supplemental Table 2. Diagnosis and staging of AKI based on Kidney Disease: Improving Global Outcomes (KDIGO) criteria (CUH)**

| AKI Stages | Serum creatinine | Urine output | Serum creatinine +  urine output | Total |
| --- | --- | --- | --- | --- |
| Stage 1 | 4 | 1 | 1 | 6 |
| Stage 2 | 5 | 2 | 10 | 17 |
| Stage 3 | 3 | 3 | 11 | 17 |
| Total | 12 | 6 | 22 | 40 |

**Supplemental Table 3: Logistic Regression Outcome AKI - CUH**

| Variables | OR | 95% C.I. | p-value |
| --- | --- | --- | --- |
| Gestational age | 1.103 | 0.822-1.481 | 0.514 |
| Gender-Male | 1.699 | 0.394-7.328 | 0.477 |
| Race-Black | 0.391 | 0.090-1.702 | 0.211 |
| Apgar score at 5min | 1.375 | 0.893-2.115 | 0.148 |
| Vasopressors | 9.015 | 1.331-61.063 | 0.024 |
| Vancomycin | 1.273 | 0.123-13.173 | 0.840 |
| Sepsis | 0.679 | 0.139-3.319 | 0.632 |

**Supplemental Table 4: Logistic Regression Outcome Mortality - CUH**

| Variables | OR | 95% C.I. | p-value |
| --- | --- | --- | --- |
| Gestational age | 1.041 | 0.786-1.378 | 0.781 |
| Gender-Male | 1.552 | 0.344-7.005 | 0.568 |
| Race-Black | 2.332 | 0.605-8.996 | 0.219 |
| Apgar score at 5min | 0.964 | 0.626-1.483 | 0.867 |
| Chorioamnionitis | 5.341 | 0.422-67.577 | 0.196 |
| Antenatal steroids | 1.269 | 0.069-23.471 | 0.873 |
| Vasopressors | 16.310 | 1.335-199.293 | 0.029 |
| Vancomycin | 0.810 | 0.065-10.174 | 0.871 |
| Sepsis | 1.255 | 0.243-6.478 | 0.786 |
| Acute Kidney Injury | 1.664 | 0.376-7.363 | 0.502 |

**Results from RWJ:**

Among 39 infants diagnosed with NEC, 24 met inclusion criteria, and 15 were excluded due to NEC staging <2. Among 24 eligible infants with NEC,16 (66.7%) had AKI of any stage, and 8 (33.3%) did not have AKI. Three (18.8%) had stage 1, 6(37.5%) had stage 2, and 7(43.8%) had stage 3 AKI. **Supplemental Table 5** compares the infants with NEC with or without AKI. The two groups had no statistically significant difference except for the mortality rate. Among 16 infants with NEC who had AKI, 8 (50%) died, whereas there was no death among the eight infants with NEC who did not have AKI (p=0.022; **Supplemental Table 5**). The median onset of NEC was 15 days, and the median AKI onset was three days prior to the onset of NEC (interquartile range - 0.5 to -6 days).

Among the 16 infants who had AKI, 5(31.25%) were diagnosed with AKI based on both SCr and UOP criteria, 11(68.7%) based on SCr only, and 0 based on UOP only **(Supplemental Table 6).** Duration noted for progression of AKI from stage 1 to 3 was 4-7 days.

Logistic regression analysis for AKI outcome showed no significant difference between the groups **(Supplemental Table 7).** There was not enough data to do logistic regression analysis for the mortality outcome.

**Supplemental Table 5. Characteristics of infants with NEC, with and without AKI in RWJ cohort**

| Variables | NEC without AKI  N=8 | NEC with AKI  N=16 | p-value |
| --- | --- | --- | --- |
| **Gestational age (mean, SD)** | 25.91(1.99) | 26.61(2.16) | 0.453 |
| **Male Sex (n, %)** | 3(37.5) | 8(50) | 0.679 |
| **Black race (n, %)** | 4(50) | 5(31.3) | 0.412 |
| **Birth weight (mean, SD)** | 688.75(128.86) | 845.31(235.84) | 0.096 |
| **C-section (n, %)** | 8(100) | 12(75) | 0.262 |
| **APGAR at 5min <7 (n, %)** | 4(50) | 7(43.75) | 0.999 |
| **Clinical Chorioamnionitis (n, %)** | 0 | 2(12.5) | 0.536 |
| **Pregnancy-induced hypertension (n, %)** | 4(50) | 7(43.8) | 1.000 |
| **Antenatal steroids (n, %)** | 6(75) | 15(93.8) | 0.249 |
| **Age at NEC onset, median (IQR)** | 16.5(6-37.25) | 14(9.25-19.5) | 0.834 |
| **Medical NEC (stage 2) (n, %)** | 4(50) | 6(37.5) | 0.327 |
| **Surgical NEC (n, %)** | 4(50) | 10(62.5) | 0.673 |
| **Blood transfusion (n, %)** | 8(100) | 15(93.8) | 1.000 |
| **Patent Ductus Arteriosus (n, %)** | 5(62.5) | 10(62.5) | 1.000 |
| **Use of Vasopressors (n, %)** | 4(50) | 12(75) | 0.363 |
| **Sepsis (Blood culture positive) (n, %)** | 2(25) | 5(31.3) | 1.000 |
| **Gentamicin (n, %)** | 8(100) | 15(93.8) | 1.000 |
| **Vancomycin (n, %)** | 4(50) | 10(62.5) | 0.673 |
| **Length of stay in survivors, days**  **median (IQR)** | 117(83-132) | 97(84-134) | 0.574 |
| **Death (n, %)** | 0 | 8(50) | 0.022 |
|  |  |  |  |

**Supplemental Table 6. Diagnosis and staging of AKI based on Kidney Disease: Improving Global Outcomes (KDIGO) criteria (RWJ)**

| AKI Stages | Serum creatinine | Urine output | Serum creatinine  +  urine output | Total |
| --- | --- | --- | --- | --- |
| Stage 1 | 3 | 0 | 0 | 3 |
| Stage 2 | 3 | 0 | 3 | 6 |
| Stage 3 | 5 | 0 | 2 | 7 |
| Total | 11 | 0 | 5 | 16 |

**Supplemental Table 7: Logistic Regression Outcome AKI -RWJ**

| Variables | OR | 95% C.I. | p-value |
| --- | --- | --- | --- |
| Gestational age | 1.435 | 0.744-2.767 | 0.282 |
| Gender-Male | 2.234 | 0.264-18.937 | 0.461 |
| Race-Black | 0.365 | 0.035-3.847 | 0.402 |
| Apgar score at 5min | 0.839 | 0.439-1.603 | 0.595 |
| Vasopressors | 5.212 | 0.394-68.979 | 0.210 |
| Vancomycin | 1.086 | 0.124-9.492 | 0.940 |
| Sepsis | 0.423 | 0.028-6.458 | 0.536 |
